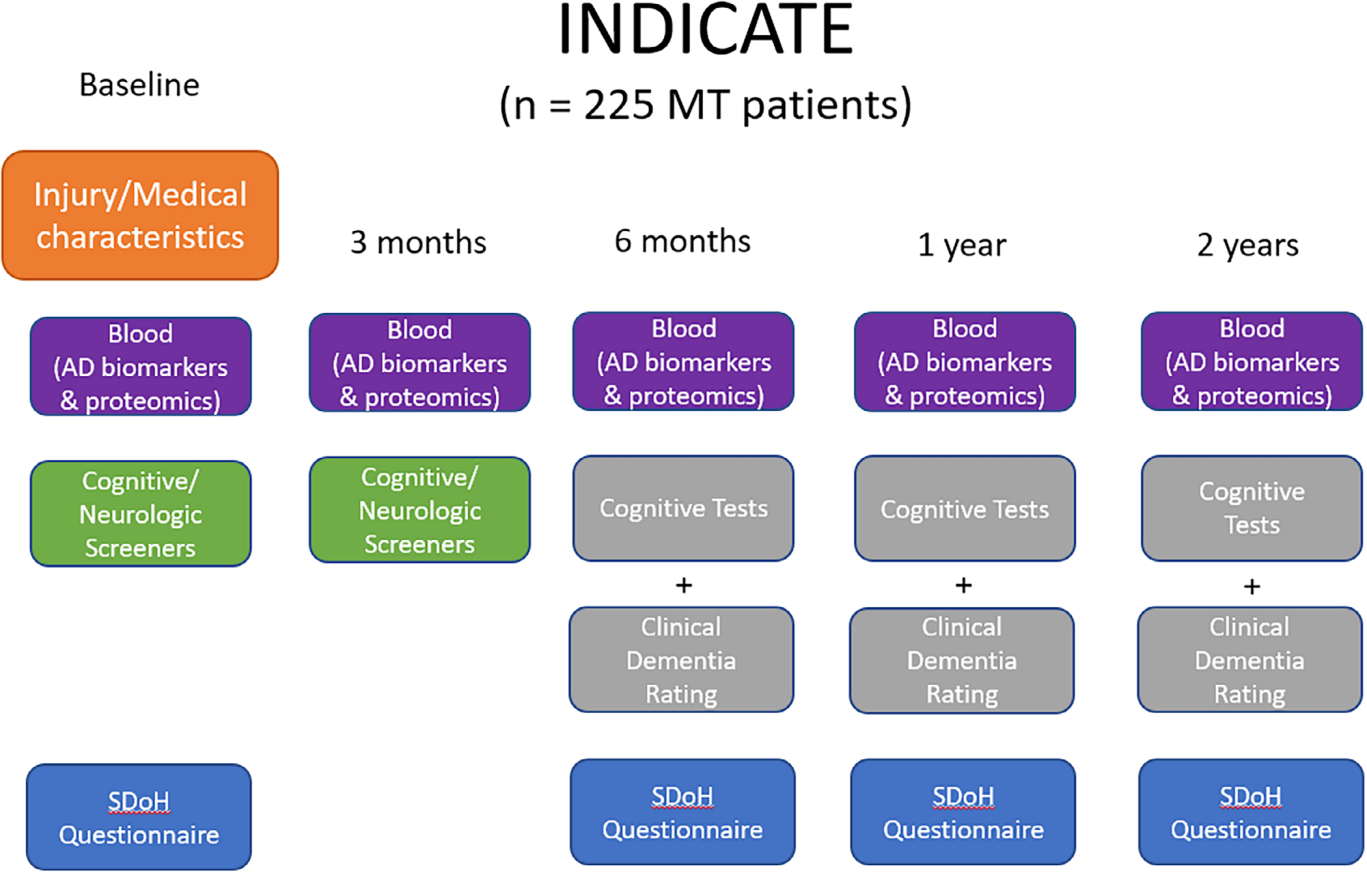# Methodology of INDICATE: Investigating Neuromarkers for Decline & Impairment of Cognition After Thrombectomy for ELVO

**DOI:** 10.1002/alz.092834

**Published:** 2025-01-09

**Authors:** Jordan P. Harp, Justin F Fraser, Christopher J McLouth, Jacqueline A Frank, Jennifer Isaacs, Elise Dahlke, Keith R Pennypacker

**Affiliations:** ^1^ University of Kentucky / Sanders‐Brown Center on Aging, Lexington, KY USA; ^2^ University of Kentucky, Lexington, KY USA

## Abstract

**Background:**

Vascular cognitive impairment and dementia (VCID) is present in 25‐30% of stroke patients, and a need exists to develop predictive biomarkers to identify patients that will incur chronic cognitive impairment. The National Plan to Address Alzheimer’s Disease and the NHLBI‐NINDS VCID Workshop called for increased research on VCID prediction, especially in underserved populations. Our stroke patient population is comprised of 70% from Appalachian areas, part of US Stroke Belt, so we are uniquely situated to perform this study. We present the design, rationale, and early implementation considerations of the INDICATE study (Investigating Neuromarkers for Decline & Impairment of Cognition After Thrombectomy for ELVO), an expansion of our novel blood and clot registry (Blood And Clot Thrombectomy Registry and Collaboration; BACTRAC).

**Method:**

The study is prospectively enrolling 225 patients, collecting systemic arterial blood at the time of thrombectomy and venous blood thereafter to analyze protein expression. Blood collections start at time of thrombectomy followed by from 3 months to 2 years post‐thrombectomy. At these time points, measures of cognition, functional status, and social determinants of health (SDoH) are also surveyed. The plasma will be analyzed for biomarkers for Alzheimer disease and related disorders (ADRD), and inflammatory proteomic biomarkers. Advanced statistical modeling will be utilized to identify biomarkers that predict functional and cognitive impairment after stroke.

**Result:**

Our group has previously identified inflammatory proteins expressed at time of thrombectomy that were predictive of functional and cognitive impairment. The study aims to validate these predictors in a broader sample with more rigorous procedures. These predictors will enable clinicians to target patients that would benefit from intensive rehabilitation.

**Conclusion:**

Capitalizing on an already existing translational model for studying stroke, we were able to design a large scale study to evaluate the relationship between ischemic stroke and cognitive outcome in human patients. The addition of ADRD biomarkers, additional time points, and more sensitive cognitive measures will strengthen our predictive model for function and cognition after stroke. We will discuss preliminary experiences with recruitment, retention, and implementation, as well as future directions.